# Personalized sepsis mortality prediction: An interpretable machine learning nomogram

**DOI:** 10.1016/j.clinsp.2026.100872

**Published:** 2026-02-15

**Authors:** Lulu Weng, Haidong Li, Yonglai Lv, Jiayi Luo, Zhenliang Wen, Jiawen Shi, Li Zhong

**Affiliations:** a Department of Critical Care Medicine, First Affiliated Hospital of Huzhou University, the First People's Hospital of Huzhou, Zhejiang, China; b Department of Spine Surgery, First Affiliated Hospital of Huzhou University, the First People's Hospital of Huzhou, Zhejiang, China; c Faculty of Engineering, Universiti Malaya, Wilayah Persekutuan Kuala Lumpur, Malaysia; d Department of Critical Care Medicine, Ruijin Hospital, Shanghai Jiao Tong University School of Medicine, China; e Department of Internal Medicine, Second People's Hospital of Anji County, Zhejiang, China

**Keywords:** Nomogram, Prediction model, Risk stratification, Intensive care unit, SHAP, Mortality prediction

## Abstract

**Purpose:**

Sepsis remains a major cause of mortality in ICU patients, requiring accurate prognostic tools for optimal management. This study aimed to develop and validate an interpretable machine learning-based nomogram for predicting in-hospital mortality in sepsis patients to guide clinical decision-making.

**Methods:**

This retrospective cohort study included 407 adult sepsis patients from ICU admissions between January 2019 and December 2024. Patients were randomly divided into training (*n* = 284) and validation (*n* = 123) cohorts. A LASSO-based multivariate logistic regression was applied to construct a predictive nomogram.

**Results:**

Seven independent predictors emerged for in-hospital mortality: age, abdominal infection, vasopressor requirement, WBC, BNP, APACHE II score, and mechanical ventilation support. The nomogram showed strong discrimination with an AUC of 0.900 in the training cohort and 0.796 in the validation cohort. Calibration curves, decision curve analysis, and SHAP interpretation confirmed good model performance and clinical utility.

**Conclusions:**

The novel machine learning-based nomogram provides a practical, interpretable risk assessment tool for early mortality prediction, potentially improving patient outcomes through enhanced clinical understanding and timely interventions in critically ill sepsis patients.

## Highlights

• A machine learning nomogram predicts in-hospital mortality in sepsis.
• Seven clinical parameters were identified as independent mortality predictors.
• The nomogram showed excellent discrimination with an AUC of 0.900.
• SHAP analysis offers interpretability to the machine learning model.
• The tool facilitates early risk stratification of high-risk patients.

**Abbreviations**
ICU: Intensive Care Unit
SOFA: Sequential Organ Failure Assessment
APACHE: Acute Physiology and Chronic Health Evaluation
CRRT: Continuous Renal Replacement Therapy
ECMO: Extracorporeal Membrane Oxygenation
PICC: Peripherally Inserted Central Catheter
CRBSI: Catheter-Related Bloodstream Infection
VIF: Variance Inflation Factor
ROC: Receiver Operating Characteristic
AUC: Area Under the Receiver operating Characteristic Curve
DCA: Decision Curve Analysis
LASSO: Least Absolute Shrinkage and Selection Operator
SHAP: SHapley Additive exPlanations
IQR: Interquartile range
CI: Confidence Interval
BNP: B-type Natriuretic Peptide
WBC: White Blood Cell Count.

## Introduction

Sepsis is a complex dysregulated host immune response to infection that can rapidly progress to life-threatening multi-organ dysfunction.^1^ critically compromising patient survival.^2^ The Global Burden of Disease Study^3^ estimated 48.9 million incident sepsis cases and 11 million sepsis-related deaths worldwide in 2017, accounting for 19.7 % of global mortality. In the United States, approximately 750,000 sepsis cases occur annually, resulting in approximately 215,000 deaths at a mortality rate of 28.7 %, constituting the primary cause of in-hospital mortality and incurring over $24 billion in healthcare expenditures.^3^ A national population-based study in China revealed an estimated 4.8 to 6.1 million annual hospital sepsis cases, with a mortality rate around 30.0 %.^4^ In Intensive Care Units (ICUs), the incidence of sepsis was 58 per 100,000 person-years, and mortality can escalate to 41.9 % or higher,^2^ underscoring the critical importance of early identification and timely intervention.

Sepsis prognosis involves multiple clinicopathological factors, including patient age,^5^ inflammatory biomarkers,^6^ and comorbidity burden.^7^ However, these individual parameters exhibit limited predictive accuracy for mortality risk.^8^ Recent machine learning approaches have shown promise but are constrained by poor interpretability,^9^^,^^10^ limited generalizability, or inadequate representation of diverse patient populations. For example, Perng et al.'s^9^ convolutional neural network model applied to 42,220 emergency patients with suspected infection achieved high accuracy but suffered from poor interpretability. In another study, Hou et al.^10^ the XGBoost model, developed using the MIMIC-III database to predict 30-day mortality in ICU sepsis patients, was limited by data deficiencies and a predominantly Caucasian patient population.

Therefore, the demand for an effective, interpretable, and clinically applicable prognostic tool for adult sepsis patients remains unmet. To address this research gap, the present study aims to develop and validate an interpretable nomogram that integrates multidimensional clinical and biological markers to predict mortality in adult ICU sepsis patients, enabling timely identification of high-risk patients and facilitating evidence-based clinical decision-making.

## Material and methods

### *Patients and study design*

This retrospective cohort study analyzed sepsis episodes in adult patients (aged ≥18 years) admitted to the First People's Hospital of Huzhou from January 2019 through December 2024. The Ethics Committee of the First Affiliated Hospital of Huzhou University approved the study protocol (approval number: 2024KYLL070–01) and waived informed consent due to the retrospective nature of the research.

**Fig. 1:**
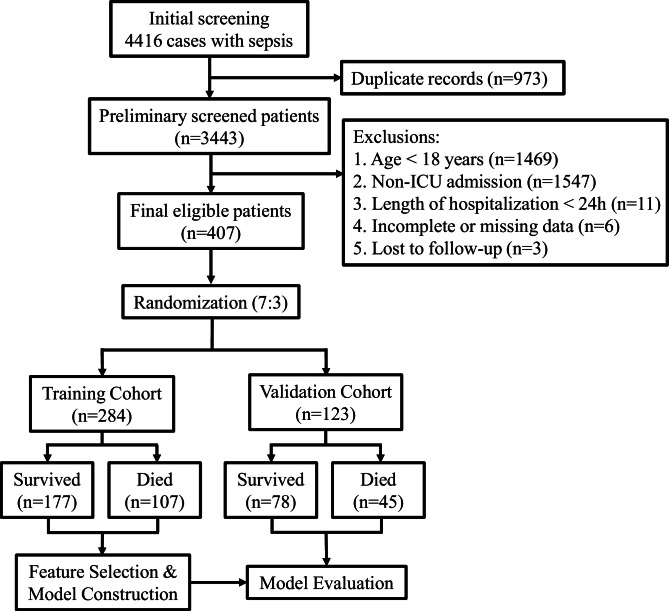
Flowchart of study participant enrollment. ICU, Intensive Care Unit.

The inclusion criterion was patients meeting the Sepsis 3.0 criteria,^1^ defined as a suspected infection combined with an acute increase of ≥ 2 points in the Sequential Organ Failure Assessment (SOFA) score. Of 4416 initially identified sepsis cases from the present hospital's electronic medical record system, 973 cases were excluded as duplicate records, leaving 3443 unique patients for preliminary screening. Consequently, 3036 patients were excluded based on the following criteria: age < 18 years (*n* = 1469), non-ICU admission (*n* = 1547), hospitalization < 24 hours (*n* = 11), incomplete data (*n* = 6), and loss to follow-up (*n* = 3). The final analysis comprised 407 patients meeting all inclusion criteria (Fig. 1).

Using R software (version 4.4.1), patients were randomly allocated to training and validation cohorts in a 7:3 ratio. The training cohort (*n* = 284; 177 survivors, 107 non-survivors) was used to construct the predictive nomogram, while the validation cohort (*n* = 123; 78 survivors, 45 non-survivors) served to internally validate the model.

### *Data collection*

A researcher blinded to the study objectives retrospectively extracted clinical data from electronic medical records. Subsequent verification was carried out by a second independent researcher to ensure data accuracy. In cases of disagreement regarding the diagnosis of sepsis or the cause of death, a third senior physician served as the adjudicator to make the final determination. Patient survival status was verified through electronic health records and the present institutional patient registry system with periodic follow-up contact. In-hospital mortality was confirmed using discharge records, death certificates, and medical documentation. Overall survival time was calculated from admission to death or last follow-up. Variables collected included demographic data, clinical parameters at sepsis onset, and laboratory measurements. Demographic variables comprised age, gender, underlying diseases, and admission type. Clinical parameters included temperature, respiratory rate, blood pressure, and heart rate. Laboratory measurements encompassed complete blood count, coagulation profile, biochemical parameters, cardiac biomarkers, inflammatory markers, and electrolytes. All clinical parameters and laboratory indices were obtained from the first 24 hours after sepsis onset. Disease severity was quantified using SOFA and Acute Physiology and Chronic Health Evaluation II (APACHE II) scores calculated from the worst values within 24 hours of sepsis onset. The dataset included infection characteristics such as site and causative microorganisms, and comprehensive therapeutic interventions. These interventions comprised invasive mechanical ventilation, Continuous Renal Replacement Therapy (CRRT), Extracorporeal Membrane Oxygenation (ECMO), Peripherally Inserted Central Catheter (PICC), vasoactive agents, antimicrobials, anticoagulants, and drainage procedures. Critical clinical outcomes included hospitalization duration, medical expenses, and in-hospital mortality.

### *Definitions*

The Sepsis-3 criteria^1^ served as the diagnostic basis for sepsis and septic shock identification. Pathogenicity of common skin flora ‒ typically coagulase-negative Staphylococcus, Bacillus species, and Corynebacterium species ‒ was established when these organisms appeared simultaneously in two separate blood cultures (obtained either consecutively from identical sites or from different anatomical locations) while patients presented clinical signs including fever (> 38.0 °C), chills, or hypotension.^11^ Catheter-Related Bloodstream Infection (CRBSI) was defined in accordance with the Clinical Practice Guidelines for the Diagnosis and Management of Intravascular Catheter-Related Infection.^12^ Immunosuppression was defined as any of the following conditions: chemotherapy/radiotherapy within 30 days pre-culture, solid organ/hematopoietic stem cell transplantation within 30 days pre-culture, or corticosteroid therapy (prednisone equivalent ≥ 25 mg daily for > 1 month or cumulative dose > 700 mg within 3 months before sepsis onset).^13^ Fluid resuscitation was defined as a crystalloid infusion of ≥ 30 mL/kg within 24 hours of sepsis diagnosis.^14^

### *Statistical analyses*

Statistical analyses were performed using SPSS version 27.0 (IBM Corp, Armonk, NY, USA), R version 4.4.1 (R Foundation for Statistical Computing, Vienna, Austria), and Python version 3.13.3 (Python Software Foundation, Beaverton, OR, USA) with statistical significance defined as a two-tailed *p* < 0.05. The choice of statistical tests was rigorously justified through diagnostic checks. For continuous variables, normality was assessed using the Shapiro-Wilk test. Variables meeting normality assumption (*p* ≥ 0.05) were further tested for homogeneity of variances using Levene's test. Those satisfying both normality and homogeneity assumptions were compared using the independent Student's *t*-test and presented as mean ± Standard Deviation. Non-normally distributed variables or those violating variance homogeneity were analyzed using the non-parametric Mann-Whitney *U* test and reported as median (interquartile range). Categorical variables, expressed as numbers (percentages), were compared using chi-square test, with Fisher's exact test applied when expected cell counts were < 5. Patients with over 10 % cumulative missing data across all study variables were excluded from the analysis. ^15^ The remaining missing data were handled via Maximum Likelihood multiple imputation using SPSS, generating five imputed datasets. The remaining missing data were addressed using Maximum Likelihood multiple imputation in SPSS, resulting in five imputed datasets. These five datasets were then combined into a comprehensive dataset according to Rubin's rules to address the missing data. Multicollinearity was evaluated using the variance inflation factor (VIF < 5 indicating acceptable collinearity) . ^16^ Variable selection utilized univariate analysis (*p* < 0.05) and Least Absolute Shrinkage and Selection Operator (LASSO) regression, with selected variables entered into multivariate logistic regression to adjust for potential confounders. A predictive nomogram was constructed from identified independent risk factors to assess the in-hospital prognosis of ICU patients. Model performance was comprehensively evaluated in both training and validation cohorts using multiple metrics, including the area under the Receiver Operating Characteristic Curve (ROC), calibration plots, and Decision Curve Analysis (DCA). SHapley Additive exPlanations (SHAP) analysis quantified predictor contributions, providing insights into their clinical impact and potential intervention thresholds.

## Results

### *Baseline and clinical characteristics of patients*

The study included 407 sepsis patients (284 training, 123 validation cohorts). Baseline characteristics were comparable between cohorts (*p* > 0.05 for all except fluid resuscitation), supporting model validity (Table 1). The median age was 72 years with male predominance. Hypertension and diabetes were prevalent comorbidities, and respiratory tract infections were the primary infection source. In the training set, non-survivors had significantly higher rates of cardiac and cerebrovascular comorbidities than survivors. In the entire cohort, non-survivors also exhibited markedly elevated APACHE II and SOFA scores and required more intensive interventions (vasopressor support, mechanical ventilation, renal replacement therapy) compared with survivors.

**Table 1:** Baseline characteristics of the patients with training and validation cohort.

**Characteristics**	**Training cohort**				**Validation cohort**			
	**Total (*n* = 284)**	**Survivors (*n* = 177)**	**Non-survivors (*n* = 107)**	**p-value**	**Total (*n* = 123)**	**Survivors (*n* = 78)**	**Non-survivors (*n* = 45)**	**p-value**
Age, median years (IQR)	72.00 (61.00, 81.00)	70.00 (58.00, 77.00)	77.00 (68.50, 84.00)	**<0.001**	73.00 (59.50, 82.00)	72.00 (55.25, 81.00)	73.00 (65.00, 82.00)	0.26
BMI, Kg/m^2^ (IQR)	21.26 (17.98, 25.04)	21.27 (18.26, 25.14)	21.01 (17.58, 24.60)	0.69	20.20 (15.56, 24.17)	20.31 (14.86, 23.78)	20.10 (15.80, 24.21)	0.85
Male sex, n ( %)	184 (64.79)	108 (61.02)	76 (71.03)	0.09	90 (73.17)	58 (74.36)	32 (71.11)	0.70
Exposure, n ( %)								
Smoking history	62 (21.83)	41 (23.16)	21 (19.63)	0.48	37 (30.08)	26 (33.33)	11 (24.44)	0.30
Alcohol drinking history	44 (15.49)	31 (17.51)	13 (12.15)	0.23	18 (14.63)	12 (15.38)	6 (13.33)	0.76
Abdominal compartment syndrome	72 (25.35)	47 (26.55)	25 (23.36)	0.55	31 (25.20)	17 (21.79)	14 (31.11)	0.25
Trauma	10 (3.52)	6 (3.39)	4 (3.74)	>0.99	7 (5.69)	4 (5.13)	3 (6.67)	>0.99
Underlying disease, n ( %)								
Hypertension	167 (58.80)	101 (57.06)	66 (61.68)	0.44	70 (56.91)	44 (56.41)	26 (57.78)	0.88
Diabetes mellitus	93 (32.75)	62 (35.03)	31 (28.97)	0.29	29 (23.58)	17 (21.79)	12 (26.67)	0.54
Bronchial asthma	6 (2.11)	1 (0.56)	5 (4.67)	0.06	5 (4.07)	3 (3.85)	2 (4.44)	>0.99
Chronic cardiac dysfunction	48 (16.90)	22 (12.43)	26 (24.30)	**0.01**	25 (20.33)	14 (17.95)	11 (24.44)	0.39
Chronic obstructive pulmonary disease	15 (5.28)	5 (2.82)	10 (9.35)	**0.02**	9 (7.32)	6 (7.69)	3 (6.67)	>0.99
Chronic renal insufficiency	30 (10.56)	15 (8.47)	15 (14.02)	0.14	9 (7.32)	5 (6.41)	4 (8.89)	0.88
Chronic hepatic insufficiency	39 (13.73)	27 (15.25)	12 (11.21)	0.34	14 (11.38)	11 (14.10)	3 (6.67)	0.21
Coronary heart disease	51 (17.96)	21 (11.86)	30 (28.04)	**<0.001**	22 (17.89)	10 (12.82)	12 (26.67)	0.05
Arrhythmia	47 (16.55)	24 (13.56)	23 (21.50)	0.08	27 (21.95)	16 (20.51)	11 (24.44)	0.61
Solid tumor	38 (13.38)	25 (14.12)	13 (12.15)	0.64	20 (16.26)	13 (16.67)	7 (15.56)	0.87
Cerebrovascular accident	58 (20.42)	25 (14.12)	33 (30.84)	**<0.001**	18 (14.63)	13 (16.67)	5 (11.11)	0.40
Peptic ulcer disease	15 (5.28)	11 (6.21)	4 (3.74)	0.37	5 (4.07)	4 (5.13)	1 (2.22)	0.76
Peripheral Vascular Disease	65 (22.89)	38 (21.47)	27 (25.23)	0.46	30 (24.39)	20 (25.64)	10 (22.22)	0.67
Suspectedsite of infection, n ( %)								
Respiratory	123 (43.31)	57 (32.20)	66 (61.68)	**<0.001**	48 (39.02)	29 (37.18)	19 (42.22)	0.58
Blood	78 (27.46)	53 (29.94)	25 (23.36)	0.23	25 (20.33)	13 (16.67)	12 (26.67)	0.18
Urinary tract	52 (18.31)	38 (21.47)	14 (13.08)	0.08	25 (20.33)	19 (24.36)	6 (13.33)	0.14
Gastrointestinal tract	15 (5.28)	10 (5.65)	5 (4.67)	0.72	4 (3.25)	2 (2.56)	2 (4.44)	0.97
Intra-abdominal	33 (11.62)	26 (14.69)	7 (6.54)	**0.04**	14 (11.38)	10 (12.82)	4 (8.89)	0.51
Intra-thoracic	7 (2.46)	5 (2.82)	2 (1.87)	0.91	1 (0.81)	0 (0.00)	1 (2.22)	0.37
Central Venous Catheter-Associated	14 (4.93)	7 (3.95)	7 (6.54)	0.33	1 (0.81)	0 (0.00)	1 (2.22)	0.37
Skin	13 (4.58)	9 (5.08)	4 (3.74)	0.82	9 (7.32)	4 (5.13)	5 (11.11)	0.39
Other	47 (16.55)	32 (18.08)	15 (14.02)	0.37	23 (18.70)	15 (19.23)	8 (17.78)	0.84
Suspectedsite of infection ≥2	81 (28.52)	50 (28.25)	31 (28.97)	0.90	29 (23.58)	18 (23.08)	11 (24.44)	0.86
Immunocompromised, n ( %)								
Immunosuppressant therapy	9 (3.17)	8 (4.52)	1 (0.93)	0.19	2 (1.63)	2 (2.56)	0 (0.00)	0.53
Chemotherapy/radiation	22 (7.75)	16 (9.04)	6 (5.61)	0.29	7 (5.69)	4 (5.13)	3 (6.67)	>0.99
Pre-sepsis surgery, n ( %)	17 (5.99)	13 (7.34)	4 (3.74)	0.21	12 (9.76)	7 (8.97)	5 (11.11)	0.95
Blood transfusion, n ( %)								
Pre-sepsis blood transfusion, n ( %)	13 (4.58)	6 (3.39)	7 (6.54)	0.35	7 (5.69)	3 (3.85)	4 (8.89)	0.45
Albumin transfusion, n ( %)	183 (64.44)	97 (54.80)	86 (80.37)	**<0.01**	78 (63.41)	45 (57.69)	33 (73.33)	0.08
Enteral Nutrition, n ( %)	154 (54.23)	78 (44.07)	76 (71.03)	**<0.01**	61 (49.59)	34 (43.59)	27 (60.00)	0.08
Parenteral Nutrition, n ( %)	54 (19.01)	34 (19.21)	20 (18.69)	0.91	27 (21.95)	14 (17.95)	13 (28.89)	0.16
Cardiopulmonary resuscitation	23 (8.10)	5 (2.82)	18 (16.82)	**<0.01**	9 (7.32)	1 (1.28)	8 (17.78)	**0.002**
Catheterization, n ( %)								
Central venous catheter^a^	195 (68.66)	111 (62.71)	84 (78.50)	**0.005**	87 (70.73)	51 (65.38)	36 (80.00)	0.09
Hemodialysis catheter^a^	83 (29.23)	35 (19.77)	48 (44.86)	**<0.001**	28 (22.76)	13 (16.67)	15 (33.33)	**0.03**
PICC^a^	39 (13.73)	12 (6.78)	27 (25.23)	**<0.001**	19 (15.45)	10 (12.82)	9 (20.00)	0.29
Drainage tube, n ( %)								
Abdominal drainage tube	51 (17.96)	37 (20.90)	14 (13.08)	0.10	22 (17.89)	14 (17.95)	8 (17.78)	0.98
Biliary drainage tube	7 (2.46)	3 (1.69)	4 (3.74)	0.50	3 (2.44)	2 (2.56)	1 (2.22)	>0.99
Thoracic drainage tube	45 (15.85)	21 (11.86)	24 (22.43)	**0.02**	12 (9.76)	4 (5.13)	8 (17.78)	0.05
Intracranial drainage tube	7 (2.46)	3 (1.69)	4 (3.74)	0.50	5 (4.07)	2 (2.56)	3 (6.67)	0.53
Urethral catheter	245 (86.27)	143 (80.79)	102 (95.33)	**<0.001**	114 (92.68)	71 (91.03)	43 (95.56)	0.57
Life-sustaining treatments								
Vasopressor, n ( %)	208 (73.24)	107 (60.45)	101 (94.39)	**<0.001**	91 (73.98)	48 (61.54)	43 (95.56)	**<0.001**
Invasive mechanical ventilation, n ( %)	136 (47.89)	52 (29.38)	84 (78.50)	**<0.001**	56 (45.53)	23 (29.49)	33 (73.33)	**<0.001**
Invasive mechanical ventilation days (IQR)	0.00 (0.00, 8.00)	0.00 (0.00, 3.00)	6.00 (1.00, 21.00)	**<0.001**	0.00 (0.00, 6.00)	0.00 (0.00, 2.00)	3.00 (0.00, 14.00)	**<0.001**
Dialysis treatment, n ( %)	83 (29.23)	35 (19.77)	48 (44.86)	**<0.001**	28 (22.76)	13 (16.67)	15 (33.33)	**0.03**
Dialysis treatment days (IQR)	0.00 (0.00, 2.00)	0.00 (0.00, 0.00)	0.00 (0.00, 5.00)	**<0.001**	0.00 (0.00, 0.00)	0.00 (0.00, 0.00)	0.00 (0.00, 4.00)	**0.01**
ECMO, n ( %)	6 (2.11)	1 (0.56)	5 (4.67)	0.06	3 (2.44)	2 (2.56)	1 (2.22)	>0.99
ECMO days (IQR)	0.00 (0.00, 0.00)	0.00 (0.00, 0.00)	0.00 (0.00, 0.00)	**0.02**	0.00 (0.00, 0.00)	0.00 (0.00, 0.00)	0.00 (0.00, 0.00)	0.69
Fluid resuscitation, n ( %)^b^	259 (91.20)^b^	157 (88.70)	102 (95.33)	0.06	101 (82.11)^b^	63 (80.77)	38 (84.44)	0.61
APACHE II score at the onset of sepsis (IQR)	27.00 (21.75, 33.00)	24.00 (19.00, 29.00)	32.00 (27.00, 37.00)	**<0.001**	27.00 (22.00, 32.00)	26.00 (20.25, 29.00)	29.00 (25.00, 35.00)	**<0.001**
SOFA score at the onset of sepsis (IQR)	8.00 (5.75, 12.00)	7.00 (5.00, 10.00)	11.00 (7.50, 13.00)	**<0.001**	8.00 (5.50, 11.00)	7.00 (4.00, 9.00)	10.00 (8.00, 14.00)	**<0.001**
Septic shock, n ( %)	98 (34.51)	49 (27.68)	49 (45.79)	**0.002**	52 (42.28)	28 (35.90)	24 (53.33)	0.06

Notes: Bold, indicates *p* < 0.05. Plus–minus values are means ± SD.

a Pre-sepsis therapeutic interventions.

b Represents the difference between the training cohort and the validation cohort; represents *p* < 0.05. IQR, interquartile range; BMI, Body Mass Index; PICC, Peripherally Inserted Central Catheter; ECMO, Extracorporeal Membrane Oxygenation; APACHE, acute physiology and chronic health evaluation; SOFA, sequential organ failure assessment.

### *Characteristics of biological indicators in the training cohort*

Laboratory analysis revealed significant differences between survivors and non-survivors in the training cohort (Table 2). Compared to survivors, non-survivors had lower body temperatures, reduced indirect bilirubin, and elevated blood urea nitrogen. Non-survivors demonstrated markedly elevated cardiac biomarkers, including myoglobin, troponin I, creatine kinase-MB, and B-type Natriuretic Peptide (BNP). Non-survivors also exhibited lower oxygen saturation and higher lactate levels, indicating poor oxygenation. Additionally, non-survivors showed lower white blood cell and neutrophil counts, reduced hemoglobin, prolonged thrombin time, elevated sodium and chloride levels, and increased lactate dehydrogenase.

**Table 2:** Comparison of biological indicators between groups of Survivors and Non-survivors in Training Group.

**Biological indicators**	**Total (*n* = 284)**	**Survivors (*n* = 177)**	**Non-survivors (*n* = 107)**	**p-value**
Temperature ( °C) (IQR)	37.20 (36.50, 37.90)	37.30 (36.80, 38.00)	37.10 (36.00, 37.60)	**<0.001**
HR (bpm) (IQR)	104.50 (80.00, 121.00)	102.00 (80.00, 120.00)	107.00 (81.50, 124.00)	0.24
RR (bpm) (IQR)	22.00 (17.00, 27.00)	21.00 (18.00, 27.00)	23.00 (17.00, 27.00)	0.33
MAP (mmHg) (IQR)	67.00 (60.00, 96.25)	67.00 (60.00, 90.00)	67.00 (59.50, 109.00)	0.95
Procalcitonin (ng/L) (IQR)	4.79 (0.49, 23.15)	6.28 (0.55, 26.37)	2.44 (0.34, 21.11)	0.07
CRP (mg/L) (IQR)	123.91 (35.53, 227.04)	136.40 (37.71, 233.75)	109.33 (29.69, 189.33)	0.22
Liver and kidney function (IQR)				
ALT (IU/L)	27.00 (15.00, 56.00)	26.00 (15.00, 50.00)	29.00 (14.50, 71.50)	0.49
AST (IU/L)	37.00 (22.75, 83.00)	34.00 (22.00, 79.00)	44.00 (25.50, 93.50)	0.09
Albumin (g/L)	30.95 (27.00, 34.38)	31.00 (27.50, 35.00)	30.40 (26.00, 34.00)	0.11
Prealbumin (mg/L)	98.50 (59.50, 168.25)	106.00 (58.00, 169.00)	95.00 (60.50, 168.00)	0.66
TBil (μmoL/L)	16.05 (10.50, 26.35)	16.30 (10.60, 28.90)	16.00 (10.35, 23.25)	0.44
IBil (μmoL/L)	6.90 (3.88, 11.72)	7.10 (4.30, 12.60)	6.60 (3.30, 10.40)	**0.04**
DBil (μmoL/L)	7.70 (4.90, 15.85)	7.40 (4.90, 16.00)	7.70 (4.95, 15.75)	0.87
BUN (mmoL/L)	12.18 (7.48, 20.02)	11.69 (7.33, 18.66)	15.23 (8.29, 23.23)	**0.04**
SCr (μmoL/L)	142.95 (92.85, 274.77)	136.70 (89.10, 252.90)	171.60 (99.00, 312.35)	0.13
SUA (μmoL/L)	355.00 (240.75, 496.00)	357.00 (260.00, 480.00)	337.00 (216.00, 565.00)	0.60
Cardiac markers (IQR)				
Myoglobin (ng/mL)	325.82 (111.43, 973.38)	247.26 (92.72, 824.20)	510.73 (172.74, 1253.24)	**0.004**
Troponin I (ng/mL)	0.05 (0.03, 0.20)	0.04 (0.03, 0.14)	0.09 (0.03, 0.36)	**0.007**
CK-MB (ng/mL)	3.88 (1.64, 9.96)	3.40 (1.35, 9.70)	5.10 (1.90, 10.57)	**0.03**
BNP (PG/mL)	190.65 (44.35, 676.68)	137.40 (41.31, 582.39)	262.18 (60.72, 1222.51)	**0.02**
Blood routine test				
WBC (×10^9^/L) (IQR)	12.35 (6.88, 19.10)	13.10 (7.50, 20.10)	10.70 (6.10, 17.25)	**0.03**
NE (×10^9^/L) (IQR)	10.77 (5.85, 17.24)	11.48 (6.62, 18.13)	9.63 (5.22, 15.97)	**0.03**
n ( %) (IQR)	90.40 (83.90, 93.90)	90.90 (84.40, 94.10)	89.40 (83.60, 93.05)	0.10
L (×10^9^/L) (IQR)	0.57 (0.36, 1.00)	0.57 (0.36, 1.03)	0.55 (0.33, 0.93)	0.48
Hb (g/L) (mean± *S*.D.)	113.50 (94.75, 133.25)	115.00 (101.00, 136.00)	111.00 (85.50, 130.00)	**0.04**
HCT ( %) (mean± *S*.D.)	33.25 (24.48, 39.50)	33.20 (26.20, 40.10)	33.50 (23.15, 38.65)	0.40
Platelet (×10^9^/L) (IQR)	138.50 (87.75, 212.25)	149.00 (92.00, 224.00)	124.00 (80.50, 203.00)	0.19
Arterial blood gas analysis				
pH (IQR)	7.38 (7.28, 7.45)	7.38 (7.31, 7.45)	7.34 (7.21, 7.44)	0.05
PaCO_2_ (mmHg) (IQR)	32.00 (25.95, 38.30)	31.00 (26.40, 36.00)	33.20 (24.40, 42.30)	0.05
PaO_2_ (mmHg) (IQR)	94.45 (71.78, 127.25)	97.00 (76.10, 132.00)	93.00 (66.60, 123.50)	0.09
SaO_2_ ( %) (IQR)	97.85 (94.68, 99.20)	98.00 (95.50, 99.30)	97.00 (93.05, 99.00)	**0.03**
Oxygenation ratio (IQR)	298.81 (205.92, 451.52)	312.86 (230.61, 463.33)	283.81 (164.50, 422.83)	**0.04**
HCO_3_^-^(mmoL/L) (mean±*S*.D.)	18.65 (14.57, 22.22)	18.40 (15.10, 21.80)	19.60 (13.25, 23.05)	0.72
Lactate (mmoL/L) (IQR)	2.55 (1.50, 4.43)	2.40 (1.40, 3.70)	2.72 (1.75, 5.15)	**0.02**
Coagulation function				
Prothrombin time (s) (IQR)	15.05 (13.40, 17.12)	15.10 (13.40, 17.10)	14.90 (13.45, 17.50)	0.44
INR (IQR)	1.25 (1.11, 1.42)	1.25 (1.11, 1.42)	1.24 (1.12, 1.41)	0.62
Fibrinogen (g/L) (mean±*S*.D.)	4.39 (3.29, 5.97)	4.64 (3.52, 6.03)	4.14 (2.88, 5.79)	0.08
APTT (s) (IQR)	34.10 (30.50, 40.00)	33.70 (30.00, 38.90)	35.00 (31.25, 42.80)	0.06
Thrombin time (s) (IQR)	15.85 (14.70, 17.33)	15.50 (14.60, 16.70)	16.40 (15.40, 18.45)	**<0.001**
D-dimer (ng/mL) (IQR)	4.10 (1.88, 9.41)	4.52 (1.87, 9.80)	3.57 (1.90, 8.47)	0.50
BG (mmoL/L) (IQR)	8.09 (5.92, 11.00)	8.25 (6.06, 10.68)	8.04 (5.36, 12.03)	0.60
Na^+^(mmoL/L) (IQR)	137.20 (133.85, 141.70)	136.40 (133.00, 140.30)	139.60 (134.70, 144.15)	**<0.001**
K^+^(mmoL/L) (IQR)	4.01 (3.50, 4.57)	3.94 (3.46, 4.45)	4.18 (3.56, 4.67)	0.07
Cl^-^ (mmoL/L) (IQR)	105.05 (99.70, 109.50)	104.40 (99.40, 108.60)	106.30 (100.65, 111.50)	**0.02**
Ca^2+^(mmoL/L) (IQR)	2.01 (1.91, 2.15)	2.02 (1.91, 2.14)	2.01 (1.90, 2.16)	0.95
Amylase (IU/L) (IQR)	60.30 (38.73, 113.20)	54.10 (36.80, 104.50)	69.70 (42.35, 133.95)	0.08
LDH (IU/L) (IQR)	287.00 (220.00, 426.75)	265.00 (205.00, 359.00)	337.00 (263.00, 504.50)	**<0.001**
CK (IU/L) (IQR)	168.00 (62.75, 476.75)	162.00 (62.00, 471.00)	177.00 (64.50, 496.00)	0.55

Notes: Clinical parameters and laboratory indices were systematically documented within 24 -hours after the onset of sepsis. Bold, indicates *p* < 0.05.

IQR, Interquartile Range; HR, Heart Rate; RR, Respiratory Rate; MAP, Mean Arterial Pressure; CRP, C-Reactive Protein; ALT, Alanine Aminotransferase; AST, Aspartate Aminotransferase; TBil, Total Bilirubin; IBil, Indirect Bilirubin; DBil, Direct Bilirubin; BUN, Blood Urea Nitrogen; SCr, Serum Creatinine; SUA, Serum Uric Acid; CK-MB, Creatine Kinase MB; BNP, Brain Natriuretic Peptide; WBC, White Blood Cell Count; NE, Neutrophilic Granulocytes; N, Percentages of Neutrophils; L, Lymphocyte count; Hb, Hemoglobin; HCT, Hematocrit; pH, Potential of Hydrogen; PaCO_2_, Partial pressure of arterial Carbon Dioxide; PaO_2_, Partial pressure of arterial Oxygen; SaO_2_, Arterial Oxygen Saturation; HCO^3-^, Bicarbonate ion; INR, International Normalized Ratio; APTT, Activated Partial Thromboplastin Time; BG, Blood Glucose; Na^+^, Sodium; K^+^, Potassium; Cl^-^, Chloride; Ca^2+^, Calcium; LDH, Lactate Dehydrogenase; CK, Creatine Kinase.

### *Characteristics of microorganisms in the training cohort*

Gram-positive organisms accounted for 56.0 % of infections, predominantly Staphylococcus species (35.9 %). Enterococcus species, particularly Enterococcus faecium, were significantly more common in non-survivors than survivors. Gram-negative organisms were found in 62.3 % of patients, mainly Enterobacteriaceae (46.8 %). *Escherichia coli* was more prevalent in survivors, while Pseudomonas aeruginosa, Stenotrophomonas maltophilia, and Acinetobacter baumannii were more common in non-survivors. Fungal infections occurred in 35.2 % of patients, with non-Candida albicans species more frequent in non-survivors. Non-survivors also had higher rates of mixed infections (three or more pathogens) and antibiotic-resistant pathogens (all *p* < 0.05) (Supplementary Table 1).

### *Characteristics of clinical outcomes in the overall cohort*

Significant differences were observed in multiple clinical outcome parameters between the two groups. Non-survivors exhibited significantly longer total ICU stay and ICU stay after sepsis, shorter hospitalization duration after sepsis and overall survival time, and substantially higher hospitalization costs. These findings underscore the severity of clinical outcomes in non-survivors (Supplementary Table 2).

### *Development of a prediction nomogram*

**Fig. 2:**
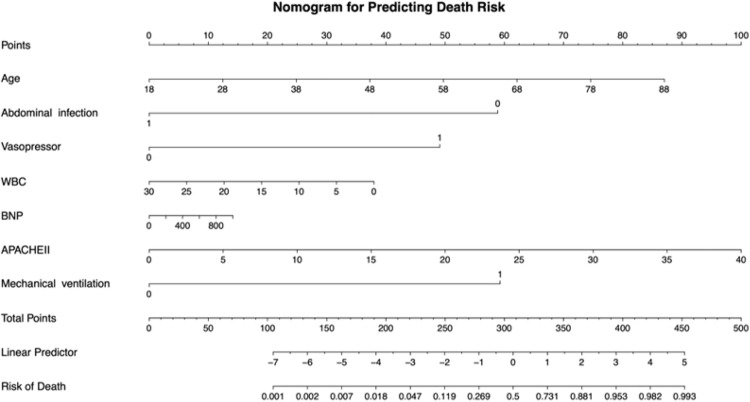
Nomogram for predicting in-hospital mortality risk in the training cohort. This nomogram integrates seven predictors: age, abdominal infection, vasopressor use, WBC, BNP, APACHE II score, and Mechanical ventilation. For each predictor, draw a vertical line to the Points scale to determine the assigned score. Summing these scores gives the Total Points, which corresponds to an estimated risk of in-hospital mortality indicated on the Risk of Death scale. The Linear Predictor scale reflects the weighted combination of predictors according to their regression coefficients, providing the mathematical basis for the nomogram's risk calculation. WBC, White Blood Cell Count; BNP, B-type Natriuretic Peptide; APACHE II, Acute Physiology and Chronic Health Evaluation II.

In this study, the authors comprehensively evaluated 48 potential prognostic features using LASSO regression with cross-validation on the training cohort to develop a non-survivors nomogram. The LASSO variable selection process inherently controls for confounding factors by identifying the most clinically significant predictors while shrinking less relevant variables toward zero. Through cross-validation, the authors determined the optimal tuning parameters, with Lambda.min at 0.01 and Lambda.1se at 0.05. At Lambda.min, 17 variables with non-zero coefficients were retained, whereas Lambda.1se yielded 7 variables (Supplementary Fig. 1). Given the superior predictive performance of the Lambda.min approach in the training and validation cohorts, the authors proceeded with the 17 selected variables for further analysis. The authors then performed bidirectional stepwise multivariate logistic regression on these 17 variables to optimize the final model, which identified 7 key predictors for nomogram construction. Multivariate logistic regression analyses revealed that age, abdominal infection, vasopressor, mechanical ventilation, White Blood Cell Count (WBC), BNP, and APACHE II were independent risk factors for non-survivors (Table 3). These variables were incorporated into a nomogram to predict post-discharge mortality (Fig. 2). To demonstrate the nomogram's utility, the authors present a hypothetical 70-year-old patient with the following characteristics: age (64-points), no abdominal infection (58-points), vasopressor use (50-points), WBC of 20×10^9/L (12-points), BNP of 200 pg/mL (3-points), APACHE II of 25 (62-points), and mechanical ventilation (60-points). The total nomogram score of 309-points suggests a 50.0 % mortality probability.

**Table 3:** Risk factors for non-survivors: results from univariate and LASSO-based multivariate regression analyses.

**Variables**	**Univariate analysis**	**p-value**	**Multivariate analysis**	**p-value**
	**OR (95% CI)**		**OR (95% CI)**	
Abdominal Infection				
No	1.00 (Reference)		1.00 (Reference)	
Yes	0.41 (0.17, 0.97)	**0.04**	0.14 (0.04, 0.43)	**<0.001**
Pulmonary Infection				
No	1.00 (Reference)			
Yes	3.39 (2.05, 5.60)	**<0.001**		
Coronary Heart Disease				
No	1.00 (Reference)			
Yes	2.89 (1.56, 5.39)	**<0.001**		
Cerebrovascular Accident				
No	1.00 (Reference)		1.00 (Reference)	
Yes	2.71 (1.50, 4.89)	**<0.001**	1.98 (0.84, 4.70)	0.12
Vasopressor				
No	1.00 (Reference)		1.00 (Reference)	
Yes	11.01 (4.58, 26.47)	**<0.001**	5.62 (1.99, 15.87)	**0.001**
PICC				
No	1.00 (Reference)			
Yes	4.64 (2.24, 9.64)	**<0.001**		
*E. coli*				
No	1.00 (Reference)			
Yes	0.42 (0.23, 0.79)	**0.007**		
CPR				
No	1.00 (Reference)			
Yes	6.96 (2.50, 19.36)	**<0.001**		
Mechanical Ventilation				
No	1.00 (Reference)			
Yes	8.78 (5.00, 15.42)	**<0.001**	6.97 (3.17, 15.31)	**<0.001**
CRRT				
No	1.00 (Reference)			
Yes	3.30 (1.94, 5.61)	**<0.001**	1.82 (0.84, 3.95)	0.13
Age	1.04 (1.02, 1.07)	**<0.001**	1.04 (1.01, 1.07)	**0.01**
Temperature	0.64 (0.51, 0.80)	**<0.001**		
WBC	0.97 (0.94, 0.99)	**0.03**	0.95 (0.92, 0.99)	**0.01**
BNP	1.01 (1.01, 1.01)	**<0.001**	1.01 (1.01, 1.01)	**0.01**
APACHEII	1.16 (1.11, 1.20)	**<0.001**	1.07 (1.02, 1.13)	**0.01**
Na^+^ (mmoL/L)	1.08 (1.03, 1.12)	**<0.001**	1.03 (0.98, 1.08)	0.20
ECMO duration	1.23 (0.93, 1.63)	0.16		
Constant			0.00 (0.00, 0.01)	**<0.001**

Notes: Variables with *p* < 0.05 in univariate analysis were included in LASSO regression (lambda.min). Variables selected by LASSO were entered into a multivariate logistic regression, and only those with *p* < 0.05 were retained. Bold indicates *p* < 0.05.

OR, Odds Ratio; CI, Confidence Interval; PICC, Peripherally Inserted Central Catheter; CPR, Cardiopulmonary Resuscitation; CRRT, Continuous Renal Replacement Therapy; WBC, White Blood Cell Count; BNP; Brain Natriuretic Peptide; APACHE, Acute Physiology and Chronic Health Evaluation; Na^+^, Sodium; ECMO, Extracorporeal Membrane Oxygenation.

### *Validation of the nomogram*

**Fig. 3:**
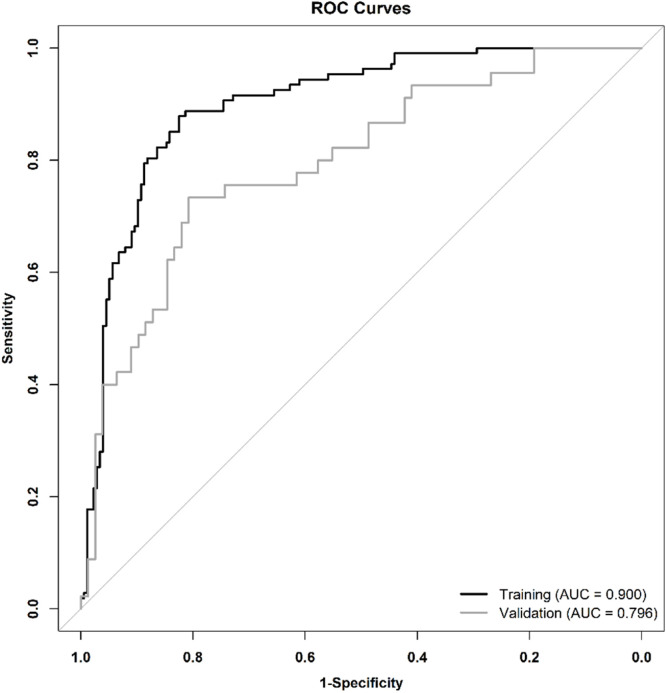
ROC curves for prediction of in-hospital mortality risk in sepsis ICU patients in training and validation cohorts. ROC, Receiver Operating Characteristic; AUC, Area Under the receiver operating Characteristic Curve.

**Fig. 4:**
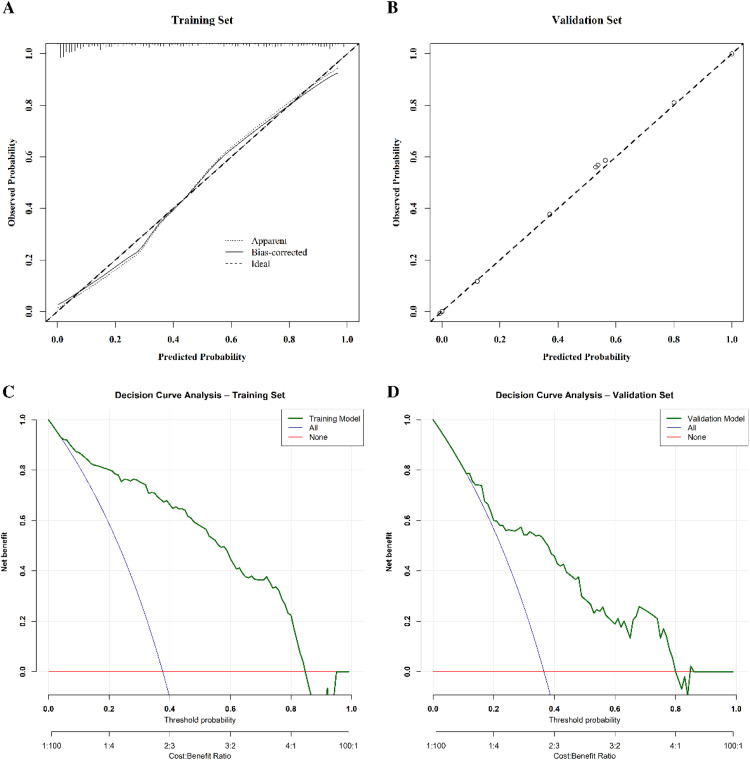
Calibration curves (A‒B) and decision curve analysis (C‒D) of the nomogram for predicting in-hospital mortality risk in sepsis ICU patients in training and validation cohorts. (A) Calibration curve for the training set. (B) Calibration curve for the validation set. The x-axis represents predicted probability, and the y-axis represents observed probability. The diagonal dashed line indicates perfect calibration. A closer fit of the calibration curve to the diagonal indicates better predictive accuracy and reliability. (C) DCA for the training set. (D) DCA for the validation set. The y-axis shows net benefit, and the x-axis represents the threshold probability. The blue line (“All”) indicates treating all patients, the red line (“None”) indicates treating none, and the green line (“Model”) indicates the net benefit of the nomogram. When the green line exceeds both the blue and red lines over most thresholds, the model offers superior clinical benefit for guiding interventions. DCA, Decision Curve Analysis.

The authors evaluated the predictive performance of the nomogram through comprehensive discriminative and calibration analyses. The nomogram showed excellent discrimination with AUCs of 0.900 (95 % CI: 0.863‒0.937) and 0.796 (95 % CI: 0.712‒0.879) in the training and validation cohorts, respectively (Fig. 3). Calibration analyses demonstrated remarkable concordance between predicted and observed probabilities across both cohorts (Fig. 4A and 4B). DCA substantiated the clinical utility of the present model in predicting ICU sepsis patients' probability of death by the time of hospital discharge, demonstrating robust predictive performance across a wide range of threshold probabilities (Fig. 4C and 4D).

### *Model interpretation using SHAP analysis*

**Fig. 5:**
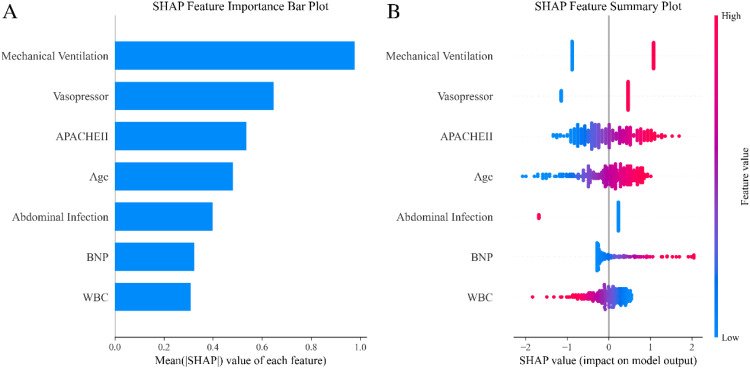
SHAP summary plots for feature importance in predicting in-hospital mortality risk in sepsis ICU patients. (A) SHAP feature importance bar plot. The mean absolute SHAP value of each feature quantifies its overall contribution to model predictions. Features are ranked by importance, with Mechanical Ventilation having the greatest impact, followed by Vasopressor use, APACHE II score, Age, Abdominal Infection, BNP, and WBC. (B) SHAP feature summary plot. Each dot represents an individual case, and the horizontal axis displays the SHAP value, which reflects both the magnitude and direction of each feature’s impact on predicted mortality risk; positive SHAP values correspond to an increased risk, while negative values correspond to a decreased risk. Dot color corresponds to actual feature values (red: high, blue: low). The distribution demonstrates both the strength and directionality of each feature's influence within the model. SHAP, SHapley Additive exPlanations; BNP, B-type natriuretic peptide; WBC, white blood cell count; APACHE II, Acute Physiology and Chronic Health Evaluation II.

The SHAP algorithm was applied to assess the importance of each predictor in relation to the risk of in-hospital mortality in ICU patients with sepsis. Fig. 5 illustrates the feature importance and summary plots, where predictors are ranked by their overall contribution to model predictions. Mechanical ventilation was identified as the most influential variable, followed by vasopressor use, APACHE II score, age, abdominal infection, BNP, and WBC. The summary plot further visualizes each variable's impact and distribution, where color represents the feature values, with red indicating higher values and blue indicating lower values.

**Fig. 6:**
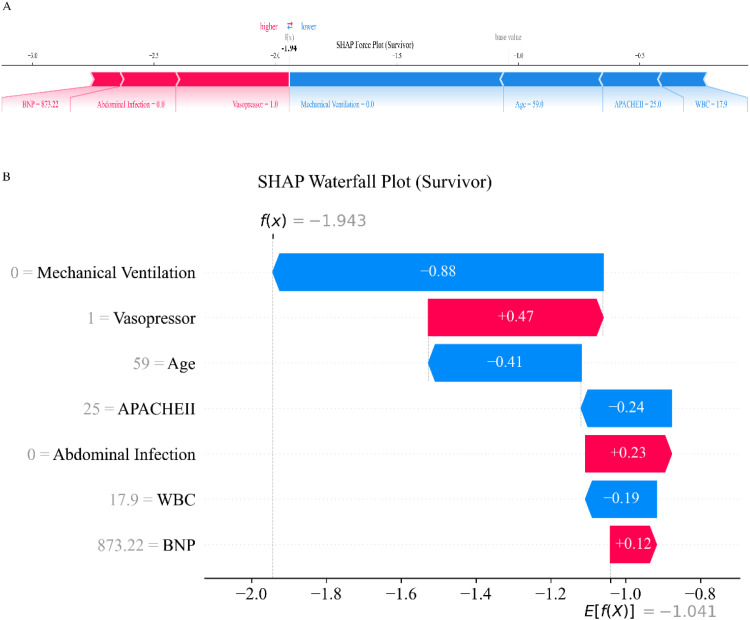
SHAP force and waterfall plots for individual prediction in a survivor case. (A) SHAP force plot visualizes how each feature contributes to the predicted in-hospital mortality risk for an individual patient. Features that increase the predicted risk are shown in red, while features that decrease the risk are shown in blue. The overall SHAP value for each feature is indicated along the horizontal axis, with the final prediction positioned relative to the mean model output (base value). (B) SHAP waterfall plot displays the cumulative contribution of each feature to the individual prediction. Starting from the base value on the right, the plot shows how each feature shifts the risk prediction up or down, with red bars representing factors that increase risk and blue bars representing factors that decrease risk. Feature values for this patient are shown on the left, and the contribution of each feature is quantified by the length of each bar. SHAP, SHapley Additive exPlanations; BNP, B-type Natriuretic Peptide; WBC, White Blood Cell Count; APACHE II, Acute Physiology and Chronic Health Evaluation II.

**Fig. 7:**
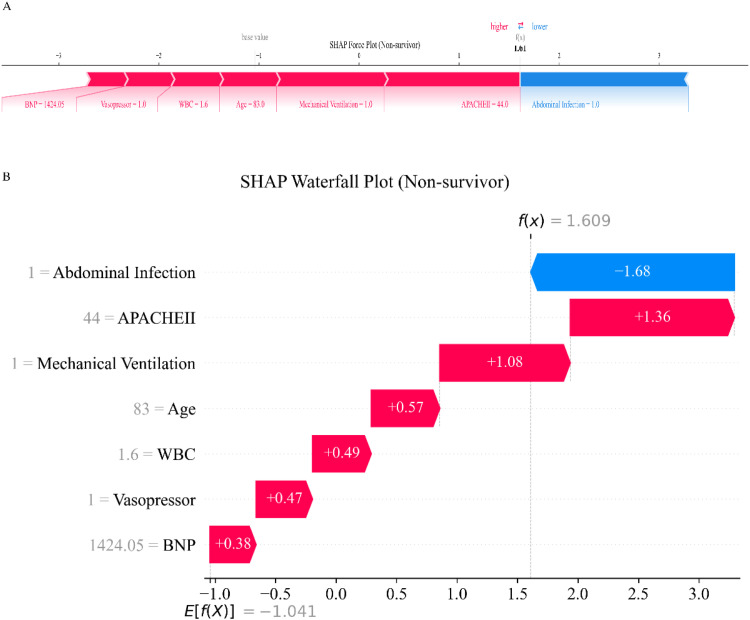
SHAP force and waterfall plots for individual prediction in a non-survivor case. (A) SHAP force plot visualizes how each feature contributes to the predicted in-hospital mortality risk for an individual patient. Features that increase the predicted risk are shown in red, while features that decrease the risk are shown in blue. The overall SHAP value for each feature is indicated along the horizontal axis, with the final prediction positioned relative to the mean model output (base value). (B) SHAP waterfall plot displays the cumulative contribution of each feature to the individual prediction. Starting from the base value on the left, the plot shows how each feature shifts the risk prediction up or down, with red bars representing factors that increase risk and blue bars representing factors that decrease risk. Feature values for this patient are shown on the left, and the contribution of each feature is quantified by the length of each bar. SHAP, SHapley Additive exPlanations; BNP, B-type Natriuretic Peptide; WBC, White Blood Cell Count; APACHE II, Acute Physiology and Chronic Health Evaluation II.

To further interpret the model’s predictions at the individual level, Fig. 6, Fig. 7 display SHAP force and waterfall plots for a survivor and a non-survivor case, respectively. In the survivor case (Fig. 6), absence of mechanical ventilation and a relatively low APACHE II score were the main contributors to a reduced risk of in-hospital mortality, as indicated by prominent negative SHAP values for these variables. Although the use of vasopressors and an elevated BNP level moderately increased risk, their effects were outweighed by factors such as younger age and absence of abdominal infection, resulting in an overall lower risk prediction. In contrast, for the non-survivor case (Fig. 7), advanced age, a high APACHE II score, and the requirement for both mechanical ventilation and vasopressors were the major drivers of elevated mortality risk, as reflected by substantial positive SHAP values. High BNP and WBC levels also contributed to increased risk. While abdominal infection was associated with a decreased predicted risk in this case, as indicated by a negative SHAP value, its contribution was insufficient to counterbalance the strong impact of adverse prognostic features, suggesting that the model is associated with a higher overall risk of in-hospital mortality.

## Discussion

By performing rigorous statistical analyses, the authors determined that age, WBC, BNP, APACHE II score, vasopressor use, and mechanical ventilation serve as independent risk factors for in-hospital mortality among ICU patients with sepsis. In contrast, the presence of abdominal infection was associated with a reduced risk of death. Utilizing these predictors, the authors developed a nomogram designed to enable personalized mortality risk assessment. This tool demonstrated excellent discriminative ability in stratifying risk for critically ill septic patients and provides a straightforward approach to inform bedside clinical decisions.

Advancing age was identified as an independent risk factor for in-hospital mortality among ICU patients with sepsis in the present study. This finding aligns with large-scale epidemiological analyses showing increased sepsis incidence and mortality in older adults.^17^ From a pathophysiological perspective, Opal et al.^18^ described immunosenescence manifestations in elderly patients, including diminished immune responses, dysregulated inflammatory mediator release, and reduced pathogen clearance capacity. These alterations not only impair defense mechanisms but also lead to atypical clinical presentations (altered mental status, less pronounced fever) that frequently cause diagnostic delays and suboptimal management,^19^ further contributing to poorer outcomes in this vulnerable population.

Lower WBC counts were strongly associated with increased in-hospital mortality in the present cohort. Consistent with the present findings, Knaus et al.^20^ demonstrated that leukopenia was associated with higher mortality in critical illness, while Georges et al.^21^ showed that patients with WBC counts < 6000 per mm^3^ had significantly increased mortality in community-acquired pneumonia. However, the relationship between WBC counts and sepsis outcomes shows conflicting patterns. Belok et al.^22^ reported complex findings in their ICU study of 5909 patients: while leukopenia was associated with higher mortality (27.2 %) compared to normal WBC counts (15.0 %), patients with leukocytosis also had elevated mortality (20.4 %), illustrating the dual risk of both WBC extremes. The Sepsis-3 consensus acknowledges this complexity by including both leukocytosis and leukopenia as indicators of sepsis-related organ dysfunction.^1^ The underlying mechanisms involve bone marrow suppression from overwhelming infection, increased peripheral leukocyte consumption, and sepsis-induced immune cell apoptosis.^23^ This compromised immune state hinders pathogen clearance, making leukopenia both a marker of immune dysfunction and a predictor of poor outcomes.

BNP emerged as a significant independent predictor of in-hospital mortality in our septic ICU cohort. As a cardiac biomarker released in response to ventricular wall stress and volume overload, ^24^ BNP reflects sepsis-induced myocardial dysfunction through multiple pathophysiological mechanisms: inflammatory mediator-induced myocardial depression,^25^ increased preload from fluid resuscitation and capillary leak, ^26^ elevated afterload secondary to vasopressor therapy,^27^ and direct upregulation by lipopolysaccharides.^28^ Post et al.^29^ showed that BNP had greater discriminative ability than APACHE II and SOFA scores for predicting 30-day mortality, while Wang and colleagues' meta-analysis^30^ revealed that BNP had a pooled sensitivity and specificity of 79.0 % and 60.0 % for mortality prediction in septic patients. However, BNP levels may be confounded by pre-existing heart failure and chronic kidney disease.^30^ Additionally, McLean et al.^31^ found that daily BNP measurements provided limited diagnostic value for predicting 28-day mortality in critically ill sepsis patients, with significant overlap between survivors and non-survivors. These findings support integrating BNP into risk stratification models, while consideration of patient-specific factors may further enhance its predictive accuracy for optimal clinical decision-making.

The APACHE II score demonstrated significant independent predictive value for in-hospital mortality in our septic cohort, confirming its well-established prognostic utility in critically ill patients. Godinjak et al.^32^ demonstrated that APACHE II had excellent discriminative ability between survivors and non-survivors in ICU patients, with sepsis/septic shock patients showing the highest predicted mortality rates. Recent evidence suggests that combining APACHE II with biomarkers such as BNP or organ dysfunction scores can enhance prognostic accuracy, ^30^ supporting our approach of integrating multiple risk factors into a comprehensive risk stratification model.

The present study identified both vasopressor use and mechanical ventilation as independent risk factors for in-hospital mortality in ICU septic patients. Multiple studies support this association: Auchet et al.^33^ examined 106 septic shock patients treated with high-dose vasopressors and reported significant mortality rates at different time points (54.2 % at 28-days and 59.8 % at 90-days), while Vincent et al.^34^ demonstrated that both cardiovascular and respiratory failures are strongly associated with adverse outcomes in critical care. The Surviving Sepsis Campaign^27^ and Sepsis-3 consensus^1^ reinforce that these interventions serve as important indicators of disease severity and prognosis. However, some evidence suggests that implementation quality may influence outcomes. Kim et al.^35^ found that early initiation of mechanical ventilation, particularly with lung-protective strategies, was associated with improved outcomes. Similarly, recent evidence indicates that early norepinephrine initiation shows potential survival benefits compared to delayed administration.^36^ While optimal implementation strategies may modify outcomes, the present results demonstrate that the utilization of these interventions remains a clinically valuable predictor of mortality risk.

An unexpected finding of the present study was the association of abdominal infection with a reduced risk of mortality. This seemingly counterintuitive result may be explained by the fact that other infection sites may confer significantly higher death rates. Prest et al.'s analysis of over 2.5 million sepsis deaths revealed striking mortality differences^37^: pulmonary sepsis demonstrated the highest rate (111.8 per-million), while abdominal sepsis showed the lowest (46.7 per-million). Motzkus et al.'s^38^ systematic review confirmed lower mortality risk for genitourinary infections compared to respiratory infections. The superior outcomes with abdominal infections likely reflect availability of definitive source control through surgical intervention, more predictable pathogen profiles, and earlier clinical recognition due to localizing symptoms.^39^ However, in contrast to the present findings, Li et al.^40^ reported higher mortality in abdominal versus pulmonary sepsis among critically ill patients with SOFA scores ≥8 in their multi-center ICU cohort. This discrepancy may be attributed to differences in study populations. The present single-center study encompassed all ICU sepsis patients irrespective of severity, whereas the increased risk for abdominal sepsis reported by Li et al. was specific to the most severely ill subgroup (SOFA ≥8). Collectively, these findings confirm that infection origin provides clinically actionable information essential for optimizing risk stratification models.

The incorporation of SHAP analysis addresses the critical “black box” limitation of machine learning approaches by decomposing individual predictions into interpretable feature contributions. Unlike conventional risk scores providing only aggregate probabilities, our SHAP visualization techniques enable comprehensive interpretability at both population and individual patient levels. This approach facilitates identification of modifiable risk factors, supports evidence-based decisions, and enhances clinical confidence in model predictions. While computational complexity may limit immediate clinical adoption, this represents a significant step toward precision medicine in sepsis management.

Several limitations should be acknowledged. First, potential selection bias due to retrospective design may limit the generalizability of the present findings. Second, uncontrolled confounding variables may exist. Although LASSO regression controlled for confounding by selecting key predictors from 48 clinical variables, residual confounding due to unmeasured factors may still exist. Third, lack of external validation or multicenter confirmation limits the present findings. While the present sample size was adequate for model development, larger multicenter datasets would enhance robustness and confirm performance across diverse clinical environments. Fourth, the single-timepoint approach challenges the model's ability to reflect sepsis's dynamic progression. Despite these limitations, the present study establishes a foundation for interpretable sepsis mortality prediction and future validation studies.

## Conclusions

In this study, the authors developed and validated an interpretable machine learning-based nomogram for predicting in-hospital mortality in ICU sepsis patients, achieving robust discrimination with AUCs of 0.900 (training cohort) and 0.796 (validation cohort). The model incorporates seven readily available clinical parameters: age, abdominal infection, vasopressor use, white blood cell count, BNP, APACHE II score, and mechanical ventilation requirement. Through SHAP analysis, this tool provides interpretable risk assessment that facilitates early identification of high-risk patients, assists in explaining individual risk factors, and supports personalized treatment decisions in clinical practice.

## Ethics approval and consent to participate

This study was approved by the Ethics Committee of the First Affiliated Hospital of Huzhou University (n° 2024KYLL070–01), and conducted according to the principles of the Declaration of Helsinki.

## Consent to publication

All authors give consent for publication.

## Availability of data and materials

All data generated or analyzed during this study are included in this manuscript and its Supplementary Materials.

## Authors’ contributions

Li Zhong: Conceptualization; methodology; resources; writing-review & editing.

Lulu Weng: Investigation; Project administration; resources; writing-original draft; writing-review & editing.

Haidong Li: Investigation; project administration; resources; writing-original draft; writing-review & editing.

Yonglai Lv: Investigation; project administration; resources; writing-original draft; writing-review & editing.

Jiayi Luo: Formal analysis; resources; writing-review & editing.

Zhenliang Wen: Formal analysis; resources; writing-review & editing.

Jiawen Shi: Investigation; project administration; resources, writing-review & editing.

## Funding

This work was supported by grants from the Medical and Health Science Program of Zhejiang Province (No. 2023KY317, L Zhong), Huzhou Municipal Science and Technology Bureau (No. 2025GYB06, L Zhong) and the Natural Science Foundation of Zhejiang Province (No. LTGD23H090001, HD Li).

## **Data availability**

The datasets generated and/or analyzed during the current study are available from the corresponding author upon reasonable request.

## Declaration of competing interest

The authors declare that they have no known competing financial interests or personal relationships that could have appeared to influence the work reported in this paper.

## Appendix Supplementary materials

Image, application 1

## References

[1] Singer M., Deutschman C.S., Seymour C.W., Shankar-Hari M., Annane D., Bauer M. (2016). The third international consensus definitions for sepsis and septic shock (Sepsis-3). JAMA.

[2] Fleischmann-Struzek C., Mellhammar L., Rose N., Cassini A., Rudd K.E., Schlattmann P. (2020). Incidence and mortality of hospital- and ICU-treated sepsis: results from an updated and expanded systematic review and meta-analysis. Intensive Care Med.

[3] Rudd K.E., Johnson S.C., Agesa K.M., Shackelford K.A., Tsoi D., Kievlan D.R. (2020). Global, regional, and national sepsis incidence and mortality, 1990-2017: analysis for the Global Burden of Disease Study. Lancet.

[4] Weng L., Xu Y., Yin P., Wang Y., Chen Y., Liu W. (2023). National incidence and mortality of hospitalized sepsis in China. Crit Care.

[5] Michels E.H.A., Butler J.M., Reijnders T.D.Y., Cremer O.L., Scicluna B.P., Uhel F. (2022). Association between age and the host response in critically ill patients with sepsis. Crit Care.

[6] Li S., Shen Y., Chang B., Wang N. (2022). Prognostic value of albumin-to-fibrinogen ratio for 28-day mortality among patients with sepsis from various infection sites. Mediat Inflamm.

[7] Rhee C., Jones T.M., Hamad Y., Pande A., Varon J., O'Brien C. (2019). Prevalence, underlying causes, and preventability of sepsis-associated mortality in US acute care hospitals. JAMA Netw Open.

[8] Makkar N., Soneja M., Arora U., Sood R., Biswas S., Jadon R.S. (2022). Prognostic utility of biomarker levels and clinical severity scoring in sepsis: a comparative study. J Investig Med.

[9] Perng J.W., Kao I.H., Kung C.T., SC Hung, Lai Y.H., Su CM. (2019). Mortality prediction of septic patients in the emergency department based on machine learning. J Clin Med.

[10] Hou N., Li M., He L., Xie B., Wang L., Zhang R. (2020). Predicting 30-days mortality for MIMIC-III patients with sepsis-3: a machine learning approach using XGboost. J Transl Med.

[11] Centers for Disease Control and Prevention (CDC) (January 2025). https://www.cdn.nhsn/pdfs/pscmanual/4psc_clabscurrent.pdf.

[12] Mermel L.A., Allon M., Bouza E., Craven D.E., Flynn P., O'Grady N.P. (2009). Clinical practice guidelines for the diagnosis and management of intravascular catheter-related infection: 2009 update by the Infectious Diseases Society of America. Clin Infect Dis.

[13] Kaech C., Elzi L., Sendi P., Frei R., Laifer G., Bassetti S. (2006). Course and outcome of Staphylococcus aureus bacteraemia: a retrospective analysis of 308 episodes in a Swiss tertiary-care centre. Clin Microbiol Infect.

[14] Levy M.M., Evans L.E., Rhodes A. (2018). The surviving Sepsis Campaign Bundle: 2018 update. Intensive Care Med.

[15] Vesin A., Azoulay E., Ruckly S., Vignoud L., Rusinovà K., Benoit D. (2013). Reporting and handling missing values in clinical studies in intensive care units. Intensive Care Med.

[16] O’brien R.M. (2007). A caution regarding rules of thumb for variance inflation factors. Qual Quant.

[17] Mayr F.B., Yende S., Angus D.C. (2014). Epidemiology of severe sepsis. Virulence.

[18] Opal S.M., Girard T.D., Ely EW. (2005). The immunopathogenesis of sepsis in elderly patients. Clin Infect Dis.

[19] Norman DC. (2016). Clinical features of infection in older adults. Clin Geriatr Med.

[20] Knaus W.A., Sun X., Nystrom O., Wagner DP. (1992). Evaluation of definitions for sepsis. Chest.

[21] Gardner J.G., Bhamidipati D.R., Rueda A.M., Nguyen D.T.M., Graviss E.A., Musher DM. (2017). White blood cell counts, alcoholism, and cirrhosis in pneumococcal pneumonia. Open Forum Infect Dis.

[22] Belok S.H., Bosch N.A., Klings E.S., Walkey AJ. (2021). Evaluation of leukopenia during sepsis as a marker of sepsis-defining organ dysfunction. PLoS One.

[23] Fuchs T.A., Abed U., Goosmann C., Hurwitz R., Schulze I., Wahn V. (2007). Novel cell death program leads to neutrophil extracellular traps. J Cell Biol.

[24] Hall C. (2004). Essential biochemistry and physiology of (NT-pro)BNP. Eur J Heart Fail.

[25] Hollenberg S.M., Singer M. (2021). Pathophysiology of sepsis-induced cardiomyopathy. Nat Rev Cardiol.

[26] Silversides J.A., Major E., Ferguson A.J., Mann E.E., McAuley D.F., Marshall J.C. (2017). Conservative fluid management or deresuscitation for patients with sepsis or acute respiratory distress syndrome following the resuscitation phase of critical illness: a systematic review and meta-analysis. Intensive Care Med.

[27] Evans L., Rhodes A., Alhazzani W., Antonelli M., Coopersmith C.M., French C. (2021). Surviving sepsis campaign: international guidelines for management of sepsis and septic shock 2021. Intensive Care Med.

[28] Tomaru Ki K., Arai M., Yokoyama T., Aihara Y., Sekiguchi Ki K., Tanaka T. (2002). Transcriptional activation of the BNP gene by lipopolysaccharide is mediated through GATA elements in neonatal rat cardiac myocytes. J Mol Cell Cardiol.

[29] Post F., Weilemann L.S., Messow C.M., Sinning C., Münzel T. (2008). B-type natriuretic peptide as a marker for sepsis-induced myocardial depression in intensive care patients. Crit Care Med.

[30] Wang F., Wu Y., Tang L., Zhu W., Chen F., Xu T. (2012). Brain natriuretic peptide for prediction of mortality in patients with sepsis: a systematic review and meta-analysis. Crit Care.

[31] McLean A.S., Huang S.J., Hyams S. (2007). Prognostic values of B-type natriuretic peptide in severe sepsis and septic shock. Crit Care Med Apr.

[32] Godinjak A., Iglica A., Rama A., Tančica I., Jusufović S., Ajanović A. (2016). Predictive value of SAPS II and APACHE II scoring systems for patient outcome in a medical intensive care unit. Acta Med Acad.

[33] Auchet T., Regnier M.A., Girerd N., Levy B. (2017). Outcome of patients with septic shock and high-dose vasopressor therapy. Ann Intensive Care.

[34] Vincent J.L., Moreno R., Takala J., Willatts S., De Mendonça A., Bruining H. (1996). The SOFA (Sepsis-related Organ Failure Assessment) score to describe organ dysfunction/failure. On behalf of the Working Group on Sepsis-Related Problems of the European Society of Intensive Care Medicine. Intensive Care Med.

[35] Kim G., Oh D.K., Lee S.Y., Park M.H., Lim CM. (Sep 9 2024). Impact of the timing of invasive mechanical ventilation in patients with sepsis: a multicenter cohort study. Crit Care.

[36] Permpikul C., Tongyoo S., Viarasilpa T., Trainarongsakul T., Chakorn T., Udompanturak S. (2019). Early use of norepinephrine in septic shock resuscitation (CENSER). A randomized trial. Am J Respir Crit Care Med.

[37] Prest J., Nguyen T., Rajah T., Prest A.B., Sathananthan M., Jeganathan N. (2022). Sepsis-related mortality rates and trends based on site of infection. Crit Care Explor.

[38] Motzkus C.A., Luckmann R. (2017). Does infection site matter? A systematic review of infection site mortality in sepsis. J Intensive Care Med.

[39] Solomkin J.S., Mazuski J.E., Bradley J.S., Rodvold K.A., Goldstein E.J., Baron E.J. (2010). Diagnosis and management of complicated intra-abdominal infection in adults and children: guidelines by the Surgical Infection Society and the Infectious Diseases Society of America. Clin Infect Dis.

[40] Li Q., Tong Y., Wang H., Ren J., Liu S., Liu T. (2021). Origin of sepsis associated with the short-term mortality of patients: a retrospective study using the eICU Collaborative Research Database. Int J Gen Med.

