# Supplementary material for: Personalized sepsis mortality prediction: An interpretable machine learning nomogram
Source: Clinics (Sao Paulo). 2026 Feb 15;81:100872. doi: 10.1016/j.clinsp.2026.100872 (PMC12924733; doi:10.1016/j.clinsp.2026.100872)

CLINICS-D-25-00909_Supplementary Material

**Supplementary Table 1** Comparison of microorganisms causing species between groups of survivors and non-survivors in training group.

| Variable [n (%)] | Total  (n=284) | Survivors  (n=177) | Non-survivors  (n=107) | *P* value |
| --- | --- | --- | --- | --- |
| Gram positive | 159 (55.99) | 100 (56.50) | 59 (55.14) | 0.82 |
| Staphylococcus | 102 (35.92) | 66 (37.29) | 36 (33.64) | 0.54 |
| Staphylococcus aureus | 34 (11.97) | 18 (10.17) | 16 (14.95) | 0.23 |
| Methicillin-resistant staphylococcus | 20 (7.04) | 12 (6.78) | 8 (7.48) | 0.82 |
| Coagulase negative staphylococcu | 74 (26.06) | 50 (28.25) | 24 (22.43) | 0.28 |
| Staphylococcus epidermidis | 29 (10.21) | 18 (10.17) | 11 (10.28) | 0.98 |
| Staphylococcus hominis | 24 (8.45) | 18 (10.17) | 6 (5.61) | 0.18 |
| Staphylococcus capitis | 12 (4.23) | 9 (5.08) | 3 (2.80) | 0.53 |
| Other coagulase negative staphylococci | 19 (6.69) | 10 (5.65) | 9 (8.41) | 0.37 |
| Enterococcus | 54 (19.01) | 27 (15.25) | 27 (25.23) | **0.04** |
| Enterococcus faecalis | 16 (5.63) | 9 (5.08) | 7 (6.54) | 0.61 |
| Enterococcus faecium | 35 (12.32) | 16 (9.04) | 19 (17.76) | **0.03** |
| Other enterococcus | 4 (1.41) | 3 (1.69) | 1 (0.93) | 0.99 |
| Streptococcus | 23 (8.10) | 18 (10.17) | 5 (4.67) | 0.10 |
| Corynebacterium | 12 (4.23) | 7 (3.95) | 5 (4.67) | >0.99 |
| Other Gram positive | 5 (1.76) | 4 (2.26) | 1 (0.93) | 0.72 |
| Gram negative | 177 (62.32) | 110 (62.15) | 67 (62.62) | 0.94 |
| Enterobacteriaceae | 133 (46.83) | 87 (49.15) | 46 (42.99) | 0.31 |
| Escherichia coli | 68 (23.94) | 52 (29.38) | 16 (14.95) | **0.006** |
| Klebsiella | 69 (24.30) | 37 (20.90) | 32 (29.91) | 0.09 |
| Klebsiella pneumoniae | 64 (22.54) | 34 (19.21) | 30 (28.04) | 0.08 |
| Other Klebsiella | 9 (3.17) | 5 (2.82) | 4 (3.74) | 0.94 |
| Other Enterobacteriaceae | 13 (4.58) | 8 (4.52) | 5 (4.67) | 1.00 |
| Proteus | 11 (3.87) | 7 (3.95) | 4 (3.74) | 1.00 |
| pseudomonas aeruginosa | 43 (15.14) | 19 (10.73) | 24 (22.43) | **0.008** |
| Stenotrophomonas maltophilia | 57 (20.07) | 23 (12.99) | 34 (31.78) | **<0.001** |
| Acinetobacter baumanii | 22 (7.75) | 8 (4.52) | 14 (13.08) | **0.009** |
| Burkholderia cepacia | 18 (6.34) | 8 (4.52) | 10 (9.35) | 0.11 |
| Haemophilus influenzae | 8 (2.82) | 5 (2.82) | 3 (2.80) | 1.00 |
| Vibrio | 6 (2.11) | 6 (3.39) | 0 (0.00) | 0.13 |
| Other Gram negative | 28 (9.86) | 17 (9.60) | 11 (10.28) | 0.85 |
| virus | 22 (7.75) | 13 (7.34) | 9 (8.41) | 0.75 |
| Fungus | 100 (35.21) | 57 (32.20) | 43 (40.19) | 0.17 |
| Candida albicans | 72 (25.35) | 44 (24.86) | 28 (26.17) | 0.81 |
| Non-candida albicans | 48 (16.90) | 22 (12.43) | 26 (24.30) | **0.01** |
| Mixed infection (≥2)^a^ | 134 (47.18) | 76 (42.94) | 58 (54.21) | 0.07 |
| Mixed infection (≥3)^b^ | 101 (35.56) | 53 (29.94) | 48 (44.86) | **0.01** |
| Pathogenic bacteria resistant to initial antibiotics | 52 (18.31) | 25 (14.12) | 27 (25.23) | **0.02** |

Notes: The time point for the biological indicators was on day of sepsis diagnosis. Bold, indicates p < 0.05.

^a^ Mixed infection refers to the presence of ≥ 2 different pathogens during the same hospitalization.

^b^ Mixed infection refers to the presence of ≥ 3 different pathogens during the same hospitalization.

**Supplementary Table 2** Comparison of clinical outcomes between Survived and Non-Survived.

| **Outcomes** | **Total**  **(n = 407)** | **Survivors**  **(n = 255)** | **Non-survivors**  **(n = 152)** | **p-value** |
| --- | --- | --- | --- | --- |
| ICU stay duration, days (IQR) | 7.00 (3.00, 16.75) | 6.00 (3.00, 12.00) | 9.00 (3.00, 24.25) | **0.03** |
| Hospital stay duration, days (IQR) | 16.00 (9.00, 30.00) | 17.00 (11.00, 28.50) | 12.00 (3.75, 35.00) | **0.006** |
| Survival time, days (IQR) | 123.00 (17.00, 427.00) | 326.00 (148.50, 715.50) | 10.00 (3.00, 28.25) | **<0.001** |
| Total hospitalization costs (IQR) | 47124.38 (23535.36, 120155.35) | 39695.66 (22538.85, 86195.68) | 82583.67 (26791.85, 175694.80) | **<0.001** |
| Average hospitalization costs (IQR) | 3046.38 (2110.98, 5350.63) | 2373.78 (1833.10, 3233.37) | 5746.19 (3863.17, 9196.96) | **<0.001** |
| ICU stay after sepsis, days (IQR) | 7.50 (3.00, 17.00) | 7.00 (3.00, 12.75) | 9.00 (3.00, 26.00) | **0.02** |
| Hospital stay after sepsis, days (IQR) | 15.00 (8.00, 28.00) | 16.00 (11.00, 27.00) | 10.50 (3.00, 29.00) | **<0.001** |

Notes: Bold, indicates p < 0.05.

ICU, Intensive Care Unit; IQR, Interquartile Range.

**Supplementary Figure 1** LASSO regression for feature selection and model tuning. (A) LASSO coefficient profiles for each predictor as a function of the logarithm of the regularization parameter (log(λ)). As λ increases, the coefficients of less important features progressively shrink toward zero and are ultimately excluded from the model, illustrating the feature selection mechanism of LASSO. (B) Ten-fold cross-validation curve used to select the optimal λ for the LASSO model. The vertical axis represents binomial deviance, and the horizontal axis displays log(λ). The dotted red line indicates λ_min, corresponding to the minimum mean binomial deviance (optimal model fit), while the dotted blue line shows λ_1se, representing the most regularized model within one standard error of the minimum. LASSO, least absolute shrinkage and selection operator; λ, regularization parameter; λ_min, minimum lambda; λ_1se, lambda at one standard error.


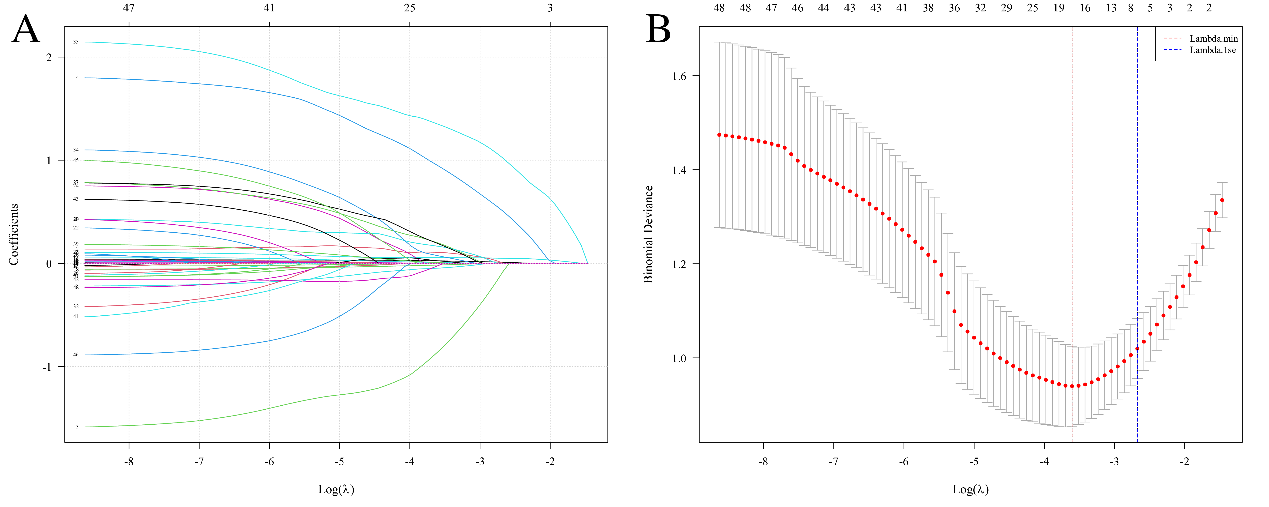

Supplement: Supplementary file 1 [file mmc1.docx]
